# Genome-Wide Analysis of *bZIP* Transcription Factors and Expression Patterns in Response to Shading Treatment in *Taxus yunnanensis*

**DOI:** 10.3390/cimb48050521

**Published:** 2026-05-17

**Authors:** Jiangtao Fan, Pengpeng Gong, Yujia Liu, Mengke Dou, Qing Li, Qiuhong Hu, Yong Wang, Gang Wang, Xiong Huang

**Affiliations:** 1 National Forestry and Grassland Southwest Engineering Technology Research Centre of Taxus, Sichuan Agricultural University, Dujiangyan 611800, China; jiangtao_fan@163.com (J.F.); 19113588485@163.com (P.G.); liuyujiashishuaige@163.com (Y.L.); dmk15836@163.com (M.D.); 19806102072@163.com (Q.L.); m17366969338@163.com (Q.H.); wangyong2015@sicau.edu.cn (Y.W.); 2 Forest Ecology and Conservation in the Upper Reaches of the Yangtze River Key Laboratory of Sichuan Province, Sichuan Agricultural University, Chengdu 610000, China

**Keywords:** *Taxus yunnanensis*, shading response, bZIP family genes, taxol biosynthesis

## Abstract

Basic leucine zipper (bZIP) transcription factors are widely involved in plant growth, development, environmental adaptation, and secondary metabolism. However, the *bZIP* gene family in *Taxus yunnanensis* has not been systematically characterized, and its potential involvement in shading-responsive regulation of paclitaxel biosynthesis remains unclear. In this study, a genome-wide analysis was performed to identify and characterize the *bZIP* family in *T. yunnanensis*. Phylogenetic analysis, conserved motif and domain identification, promoter cis-element analysis, chromosomal localization, and expression profiling were conducted to investigate their structural features and regulatory potential. A total of 18 *TyubZIP* genes were identified and classified into 10 subfamilies. These genes exhibited variation in physicochemical properties but showed conserved structural features and nuclear localization. Promoter analysis revealed abundant light-responsive, hormone-related, and stress-related cis-elements. Expression profiling indicated tissue-specific expression patterns and diverse responses to shading treatment. WGCNA further identified candidate *TyubZIP* genes potentially associated with paclitaxel biosynthesis. Among them, *TyuHY5* was selected for functional analysis. Subcellular localization and transcriptional assays demonstrated that *TyuHY5* can bind to the promoter of *TyuDBTNBT* and positively regulate its activity. These findings provide the first genome-wide characterization of the bZIP family in *T. yunnanensis* and identify *TyuHY5* as a shading-responsive candidate regulator of paclitaxel biosynthesis, providing insights that may inform the genetic improvement and cultivation strategies of *Taxus* for enhanced paclitaxel production.

## 1. Introduction

*Taxus yunnanensis,* an endemic evergreen conifer from southwestern China, is one of the most important medicinal resources within the genus *Taxus* [1]. It produces taxane diterpenoids, particularly paclitaxel, a potent antitumor compound that has attracted extensive attention in natural product research and pharmaceutical development [2]. Accumulating evidence indicates that plant secondary metabolism is highly responsive to environmental cues, with light acting as a key regulatory factor [3,4]. In Taxus species, variations in light intensity or shading conditions significantly affect paclitaxel accumulation and the activity of its biosynthetic pathway [5]. Moreover, *Taxus* species grow slowly and experience frequent light fluctuations during natural regeneration and cultivation [6]. Therefore, understanding their molecular responses to shading is essential for elucidating light adaptation and provides insights into the regulation of paclitaxel biosynthesis [7]. More broadly, light signaling regulates diverse classes of secondary metabolites, including flavonoids, terpenoids, and alkaloids, through complex transcriptional networks [8]. However, in *T. yunnanensis* and related species, transcriptional regulatory mechanisms underlying light-responsive secondary metabolism remain poorly understood, despite extensive studies on biosynthetic pathways and metabolic engineering [9]. Among plant transcription factor families, basic leucine zipper (bZIP) proteins are key regulators of light signaling, abiotic stress responses, and metabolic processes [10,11,12].

The bZIP transcription factor family is widely studied in plants and exhibits a high degree of conservation across eukaryotic species [13]. bZIP proteins possess a conserved domain composed of a DNA-binding basic region and a leucine zipper required for dimerization [14]. The basic region binds to ACGT-containing cis-elements in target promoters, including the G-box, C-box, and A-box, whereas the leucine zipper enables homo- or heterodimer formation, thereby substantially expanding their regulatory diversity [15]. Outside this conserved domain, bZIP proteins exhibit highly variable N- and/or C-terminal regions that typically function as transcriptional activation or repression domains [16]. Owing to their sequence-specific DNA-binding activity and versatile dimerization properties, *bZIP* transcription factors participate in diverse biological processes, including photomorphogenesis, hormone signaling, abiotic stress responses, and the regulation of both primary and specialized metabolism [14,17,18].

Accumulating evidence suggests that *bZIP* transcription factors are not only involved in plant growth and stress adaptation, but also play crucial roles in the light-mediated regulation of specialized metabolism [10]. Among them, ELONGATED HYPOCOTYL 5 (*HY5*) is a well-characterized *bZIP* transcription factor and a central regulator of light signaling, which has been shown to control the transcription of multiple genes involved in secondary metabolism [19,20]. In *Artemisia annua*, light-induced artemisinin biosynthesis is regulated by *AaHY5*, highlighting the direct involvement of *bZIP* members in the light-dependent accumulation of pharmaceutically important metabolites [21,22]. Moreover, *HY5* directly binds to G-box or ACE motifs in the promoters of *MYB* transcription factors such as *MYB75/PAP1* and *MYB1*, thereby activating their expression and promoting anthocyanin biosynthesis [23,24]. However, the regulatory functions of HY5-like factors in specialized metabolism are highly context-dependent across species and pathways. For instance, in *Camptotheca acuminata*, overexpression of *CaHY5* suppresses camptothecin biosynthetic gene expression and reduces metabolite accumulation in leaves [25]. In addition, recent studies in *Uncaria* species have shown that light intensity modulates the accumulation of mitragynine and isomitraphylline, while PHYTOCHROME-INTERACTING FACTOR 3 (*PIF3*)-mediated low-light signaling participates in regulating isomitraphylline biosynthesis, further emphasizing the importance of light-responsive transcriptional networks in medicinal metabolite production [26]. Collectively, these findings establish light-associated transcription factors, particularly *bZIP* members, as key regulatory hubs linking environmental light cues to specialized metabolism [27]. However, their roles in *Taxus* species, especially *T. yunnanensis*, in shading-responsive metabolic regulation remain largely unexplored [28]. Therefore, systematic characterization of the *bZIP* gene family and its expression responses under shading conditions is essential to elucidate the molecular basis of light-regulated secondary metabolism in this species.

Although the reference genome of *T. yunnanensis* has been released [2], the *bZIP* transcription factor family remains poorly characterized, and its roles in shading responses are still unclear. In this study, we conducted a genome-wide identification and comprehensive analysis of the *bZIP* gene family in *T. yunnanensis*. Gradient shading treatments combined with transcriptome sequencing were then applied to investigate expression dynamics under different light conditions. Based on expression profiling and weighted gene co-expression network analysis (WGCNA), candidate *bZIP* genes potentially involved in paclitaxel biosynthesis were identified. Among them, *TyuHY5* was selected for functional validation and was shown to directly bind to the promoter of the key paclitaxel biosynthetic gene *DBTNBT*. Collectively, these results reveal a *bZIP* mediated transcriptional regulatory network underlying shading responses in *T. yunnanensis*, providing new insights into the molecular basis of light-regulated secondary metabolism and offering potential targets for improving paclitaxel production.

## 2. Materials and Methods

### 2.1. Plant Materials and Treatments

The shading experiment was conducted in April 2024 at the experimental field of Sichuan Agricultural University, Chengdu, China (103°51′ E, 30°42′ N), using three-year-old potted seedlings of *T. yunnanensis*. The plant materials were formally identified by Professor Xiaohong Chen, and their use was approved by the Sichuan Agricultural University. All experimental plants were grown from seeds collected from a single mother plant to ensure genetic uniformity. Seedlings were cultivated in nursery containers (25 cm in diameter × 30 cm in height) filled with sandy soil. Five shading treatments were established using black nylon nets positioned 1.5 m above the canopy, corresponding to 20%, 40%, 60%, 80%, and 100% of full light (designated as T1–T5). Each shading treatment included 10 potted seedlings as biological replicates, and all plants were grown under uniform conditions. To quantify the actual light intensity under each treatment, photosynthetic active radiation (PAR; μmol m^−2^ s^−1^) at canopy level was measured. Midday natural sunlight typically reaches ~1500 μmol m^−2^ s^−1^. Based on this reference, the estimated PAR under each shading treatment was: T1 (20% shading) = 292.5 μmol m^−2^ s^−1^, T2 (40% shading) = 654.1 μmol m^−2^ s^−1^, T3 (60% shading) = 941.8 μmol m^−2^ s^−1^, T4 (80% shading) = 1223.7 μmol m^−2^ s^−1^, T5 (100% full light) = 1396.8 μmol m^−2^ s^−1^. These values provide a quantitative basis for interpreting shading-responsive transcriptional responses. All plants were maintained under uniform management conditions. After 120 days of treatment, healthy and physiologically uniform seedlings were harvested for transcriptome analysis and related experiments. Each treatment included three biological replicates.

### 2.2. RNA Sequencing and Expression Data Processing

Total RNA was extracted from each sample following the manufacturer’s instructions and assessed for quality before library construction (Tiangen Biotech Co., Ltd., Beijing, China). RNA-seq libraries were prepared using a standard Illumina protocol and sequenced on an Illumina platform (Illumina, San Diego, CA, USA) to generate paired-end reads. Raw reads were filtered to remove adapters and low-quality sequences using FastQC and Trimmomatic [29,30]. Clean reads were mapped to the reference genome of *T. yunnanensis* using HISAT2 version 2.2.1 [2,31]. Gene expression levels were quantified using featureCounts and normalized as FPKM [32]. Differential expression analysis was performed using DESeq2 with |log2FC| ≥ 1 and FDR < 0.05 as the significance threshold [33].

### 2.3. Identification of bZIP Gene Family

The genome sequence of *T. yunnanensis* was retrieved from the 1K Medicinal Plant Genome Database for genome-wide analysis (http://www.herbgenome.com/). Candidate *bZIP* transcription factors were identified using an HMM search based on the PF00170 profile obtained from the Pfam database. The HMMER 3.0 software was used with an E-value threshold of 1 × 10^−10^ to screen potential bZIP proteins [34]. Candidate genes were screened by BLASTP against known plant bZIP proteins, and conserved domains were identified using the Pfam and SMART databases [35]. Redundant sequences and incomplete gene models were removed prior to downstream analyses. Protein physicochemical parameters, including molecular weight, isoelectric point, and GRAVY values, were obtained through the ExPASy platform. Subcellular localization predictions were carried out using WoLF PSORT.

### 2.4. Phylogenetic Analysis and Classification of bZIP Proteins

Phylogenetic relationships were inferred for bZIP proteins from *T. yunnanensis* and *A. thaliana*. Arabidopsis bZIP protein sequences were retrieved from the TAIR11 database. Multiple sequence alignment was performed using ClustalW with default parameters. The phylogenetic tree was inferred using the maximum likelihood (ML) method in MEGA 7 with 1000 bootstrap replicates [36]. The resulting tree was visualized and annotated using Evolview2 [37]. Based on clustering with *A. thaliana* bZIP proteins, *T. yunnanensis* bZIP were classified into distinct subgroups according to their evolutionary relationships [15].

### 2.5. Motif and Conserved Domain Analysis of bZIP Proteins

Conserved motifs of bZIP proteins were identified using the MEME Suite with default parameters, and the maximum number of motifs was set according to the analysis requirement [38]. Identified motifs were visualized and annotated based on their distribution across the protein sequences. In addition, conserved domain architecture was analyzed to characterize functional features of bZIP proteins. Domain annotations were obtained using online conserved domain databases [39], and the distribution of conserved domains was visualized together with motif structures to compare differences among bZIP subgroups.

### 2.6. Promoter Cis-Element Analysis of bZIP Genes

The 2000 bp upstream sequences of bZIP genes were extracted as promoter regions and analyzed using PlantCARE to predict cis-regulatory elements. Identified elements were classified based on their functional annotations, including light-responsive, hormone-responsive, and stress-related elements. The distribution of cis-elements in the promoter regions was visualized to explore potential regulatory roles of bZIP genes under shading conditions [39].

### 2.7. Chromosomal Distribution and Syntenic Relationships of bZIP Genes

The chromosomal positions of *bZIP* genes were obtained from the genome annotation file and visualized based on their physical locations on each chromosome [40]. To investigate gene duplication and evolutionary relationships, synteny analysis was performed between *T. yunnanensis* and representative plant species, including *A. thaliana*, *Catharanthus roseus*, *Phellodendron amurense*, *Salvia miltiorrhiza*, and *Ginkgo biloba*. Homologous gene pairs were identified using sequence similarity searches, and collinearity relationships were analyzed using MCScanX [41].

### 2.8. Expression Analysis of bZIP Transcription Factors and WGCNA Analysis

The expression patterns of *bZIP* transcription factors were analyzed using both publicly available RNA-seq data and transcriptome datasets generated in this study. For tissue-specific analysis, RNA-seq data covering five tissues (bark, leaf, root, stem, and twig bark) were retrieved from the NCBI database (BioProject: PRJNA661543) [2]. For shading-related analysis, expression profiles generated in this study were used to assess *bZIP* gene responses to different shading treatments. WGCNA was performed to identify gene co-expression modules associated with shading treatment and the taxol biosynthetic pathway. The analysis was conducted using the WGCNA R package version 1.72-1 in R version 4.2.2 [42]. A signed network was constructed using a soft-thresholding power β = 10, selected to achieve scale-free topology with R^2^ > 0.85 (Figure S1). Modules were identified using dynamic tree cutting, and module–trait relationship analysis was subsequently performed to evaluate the association between individual modules and irradiance treatments. The tan module showed the strongest positive correlation with irradiance treatment (r = 0.85, *p* = 0.002), indicating that genes within this module may participate in light-responsive regulatory processes. Hub genes were determined according to intramodular connectivity. Within the tan module, *TyuDBTNBT*, *TyuHY5*, and several other *TyubZIP* genes exhibited relatively high connectivity (kWithin > 0.8), suggesting that they may function as central regulators in the light-responsive transcriptional network associated with paclitaxel biosynthesis.

### 2.9. Sequence Alignment, Phylogenetic Analysis, and Subcellular Localization of TyuHY5

The full-length amino acid sequence of *TyuHY5* was used for evolutionary analysis. HY5 protein sequences from multiple plant species were retrieved from the NCBI database, including *Solanum lycopersicum* (accession no. XP_069152151.1), *Ricinus communis* (accession no. XP_002519348.1), *Hevea brasiliensis* (accession no. XP_021670974.2), *A. annua* (accession no. PWA48328.1), *Uncaria rhynchophylla* (accession no. PX909993.1), *S. miltiorrhiza* (accession no. XP_057776425.1), *Dendrobium catenatum* (accession no. XP_020685894.1), and *A. thaliana* (accession no. NP_001330553.1). Multiple sequence alignment of the HY5 protein sequences was performed using ClustalW version 2.1. A phylogenetic tree was constructed using the maximum likelihood (ML) method in MEGA 7 with 1000 bootstrap replicates. The resulting tree was visualized and edited using TBtools-II v2.441 [39]. For subcellular localization analysis, the coding sequence of *TyuHY5* without the stop codon was fused to GFP in the pCAMBIA1300-35S: GFP vector. The recombinant construct was introduced into *Agrobacterium tumefaciens* strain GV3101 (Weidi Biotechnology Co., Ltd., Shanghai, China) by the freeze–thaw method and transiently expressed in 5-week-old *Nicotiana benthamiana* leaves via Agrobacterium-mediated infiltration. Fluorescence signals were observed 3 days after infiltration using a Leica confocal laser scanning microscope (Leica TCS SP8, Leica Microsystems, Wetzlar, Germany) to determine the subcellular localization of *TyuHY5* [43].

### 2.10. Yeast One-Hybrid Assays

A yeast one-hybrid (Y1H) assay was performed to examine the interaction between *TyuHY5* and the promoter of *TyuDBTNBT* [44]. The coding sequence of *TyuHY5* was cloned into the pGADT7 vector, and the promoter fragment of *TyuDBTNBT* was inserted into the pHIS2 vector. The empty pGADT7 vector co-transformed with pHIS2-TyuDBTNBT-pro was used as a negative control. All primers used in this study are listed in Table S1. The recombinant constructs were co-transformed into yeast competent cells (Weidi Biotechnology Co., Ltd., Shanghai, China) according to the manufacturer’s instructions. Transformants were first selected on SD/-Trp/-Leu medium (Coolaber Science & Technology Co., Ltd., Beijing, China) and then transferred onto SD/-Trp/-Leu/-His medium supplemented with 30, 60, and 90 mM 3-amino-1,2,4-triazole (3-AT) (Solarbio Science & Technology Co., Ltd., Beijing, China). Yeast growth on the selective medium was used to assess the interaction between *TyuHY5* and the *TyuDBTNBT* promoter.

### 2.11. Dual-Luciferase Reporter Assay

A dual-luciferase reporter assay was performed to investigate whether *TyuHY5* regulates the promoter activity of *TyuDBTNBT* [45]. The full-length coding sequence of *TyuHY5* was cloned into the pGreenII 62-SK vector as the effector construct, while the promoter fragment of *TyuDBTNBT* was inserted into the pGreenII 0800-LUC vector as the reporter construct. All primers used in this study are listed in Table S1. The recombinant plasmids were transformed into *A. tumefaciens* strain GV3101 (Weidi Biotechnology Co., Ltd., Shanghai, China). The effector and reporter constructs were co-infiltrated into leaves of *N. benthamiana*. After transient expression, luciferase signals were detected using a plant luminescence imaging system (Tanon 5200, Tanon Science & Technology Co., Ltd., Shanghai, China) following application of luciferin substrate (Beyotime Biotechnology, Shanghai, China). In addition, LUC and REN activities were quantified using a dual-luciferase reporter assay kit (Beyotime Biotechnology, Shanghai, China) according to the manufacturer’s instructions. The relative promoter activity was expressed as the ratio of firefly luciferase (LUC) to Renilla luciferase (REN).

## 3. Results

### 3.1. Identification and Basic Characterization of TyubZIP Genes

Based on the reference genome of *T. yunnanensis*, a total of 18 *bZIP* genes were identified and designated *TyubZIP01*–*TyubZIP18*. Detailed information, including gene IDs and physicochemical properties of the encoded proteins, is summarized in Table S2. The predicted TyubZIP proteins exhibited considerable variation in their physicochemical characteristics. The molecular weights ranged from 17.73 to 68.23 kDa, while the theoretical isoelectric points (pI) varied from 5.04 to 10.40, indicating substantial diversity in protein size and charge properties among family members. All TyubZIP proteins showed negative GRAVY values, ranging from −1.068 to −0.484, suggesting that they are predominantly hydrophilic. In addition, subcellular localization prediction indicated that all TyubZIP proteins are localized in the nucleus, consistent with their putative roles as transcription factors.

### 3.2. Phylogenetic Analysis of TyubZIP Proteins

**Figure 1:**
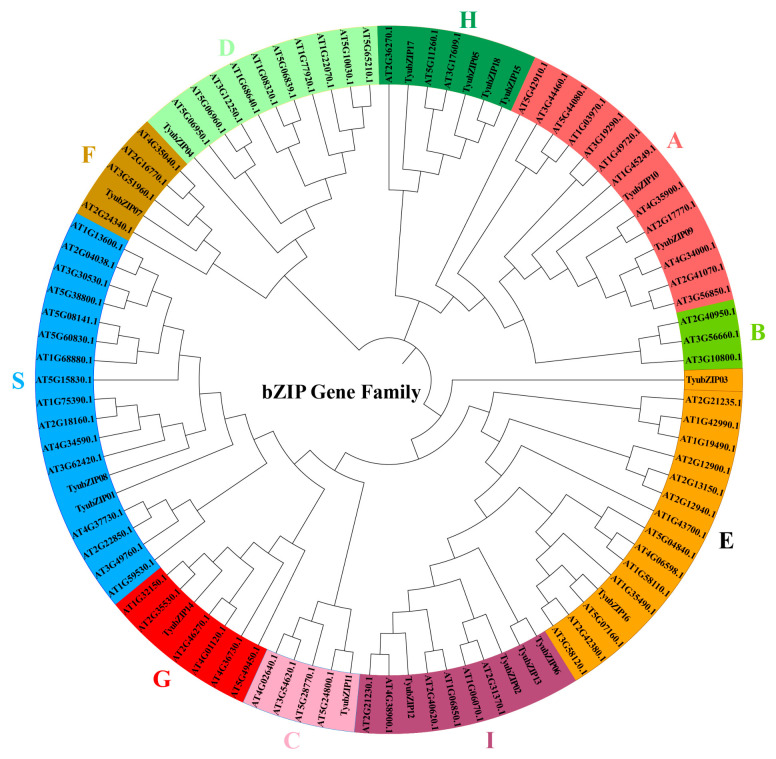
Phylogenetic analysis of bZIP proteins from *Taxus yunnanensis* and *Arabidopsis thaliana*. The bZIP proteins were classified into 10 subfamilies (A, B, C, D, E, F, G, H, I, and S) according to their phylogenetic relationships. Different colors represent different subfamilies, and TyubZIP proteins are shown in bold.

To investigate the evolutionary relationships of bZIP proteins, a phylogenetic tree was constructed based on 96 bZIP protein sequences, including 78 Arabidopsis bZIPs and 18 TyubZIPs (Figure 1). According to their phylogenetic relationships with *Arabidopsis* bZIP proteins, the TyubZIP proteins were classified into 10 subfamilies, namely A, B, C, D, E, F, G, H, I, and S, consistent with the classification reported for the plant bZIP family. The distribution of TyubZIP members among these subfamilies was uneven. No TyubZIP protein was assigned to one of the Arabidopsis-defined clades, whereas the H and I subfamilies each contained four TyubZIP members. The remaining TyubZIP proteins were distributed among the other subfamilies in smaller numbers. Notably, the four TyubZIP members in the H subfamily clustered together with AtHY5 and its closely related homologs, indicating a close evolutionary relationship between these proteins.

### 3.3. Conserved Motif and Domain Analysis of TyubZIP Proteins

**Figure 2:**
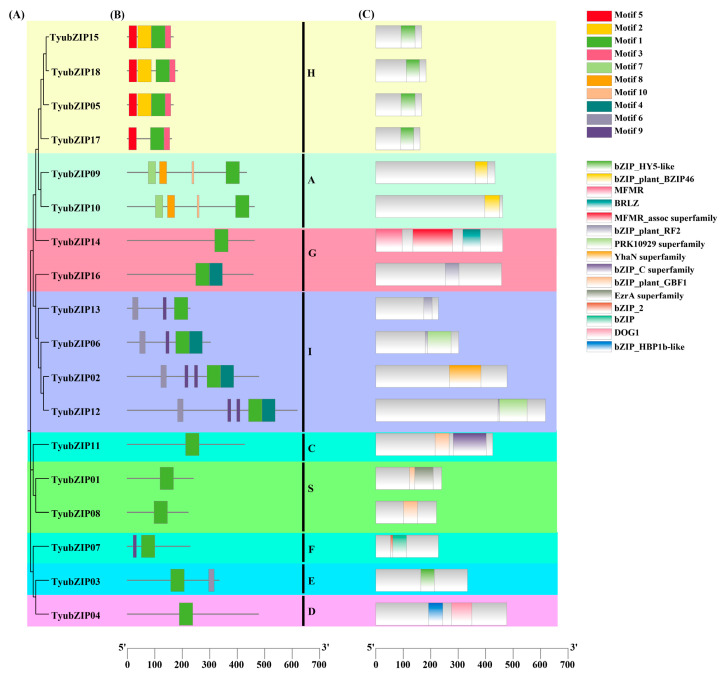
Phylogenetic relationships, conserved motif composition, and domain organization of TyubZIP proteins. The uppercase letters (H, A, G, I, C, S, F, E, and D) in the middle panel represent the corresponding bZIP subfamilies. (**A**) Phylogenetic tree of the 18 TyubZIP proteins. (**B**) Distribution of conserved motifs in TyubZIP proteins. Different colored boxes indicate different motifs, and gray lines indicate non-conserved regions. Motif 1, present in all TyubZIPs, largely overlaps with the annotated bZIP domain, corresponding to the DNA-binding and dimerization region, indicating a core conserved functional element. (**C**) Predicted conserved domain organization of TyubZIP proteins. Different colored boxes indicate different domains, and gray lines indicate non-conserved regions.

To further investigate the structural conservation and divergence of the *TyubZIP* family, the conserved motifs and domain composition of TyubZIP proteins were analyzed (Figure 2). A total of 10 conserved motifs were identified, and their sequences are listed in Table S3. Among them, Motif 1 was detected in all *TyubZIP* members and was located at a relatively conserved position, suggesting that it represents a core conserved element of TyubZIP proteins. In addition, several conserved motifs associated with the bZIP domain were mainly distributed in the C-terminal region of most TyubZIP proteins, consistent with the typical structural features of *bZIP* transcription factors. Notably, the four TyubZIP proteins identified as HY5 homologs in the phylogenetic analysis exhibited highly similar motif compositions. All four proteins contained Motifs 1, 2, 3, and 5 in a similar arrangement, forming a characteristic motif organization distinct from that of other subfamilies. Motif 1 was detected in all TyubZIP proteins, representing a core conserved element. In most TyubZIPs, Motif 1 overlaps with the annotated bZIP domain, corresponding to the DNA-binding and dimerization region, supporting its functional conservation across the family. These results indicate that the HY5-like members of the *TyubZIP* family are highly conserved at the structural level.

### 3.4. Analysis of Promoter Cis-Regulatory Element of TyubZIP Genes

**Figure 3:**
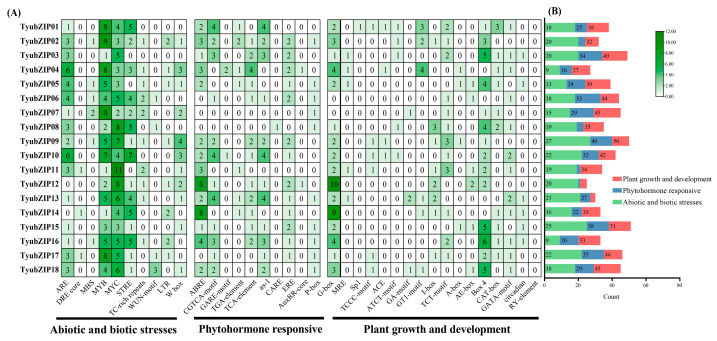
Cis-acting element analysis of *TyubZIP* promoter regions. (**A**) Distribution patterns of cis-acting elements in the promoter regions of *TyubZIP* genes. The number of each cis-element is represented by numerical values and a color gradient. (**B**) Quantitative summary of cis-acting elements grouped into different functional categories in *TyubZIP* promoters.

To investigate the potential regulatory features of *TyubZIP*, cis-acting elements in their promoter regions were analyzed (Figure 3A,B). The results showed that the promoters of *TyubZIP* contained diverse cis-acting elements related to abiotic stress responses, hormone signaling, and light responsiveness, indicating complex transcriptional regulatory potential. Among the stress- and hormone-related elements, MYB, MYC, ABRE, STRE, and TC-rich repeats were widely distributed in multiple *TyubZIP* promoters, while LTR elements were detected in several genes (Figure 3A). In addition, light-responsive elements, including G-box, Box 4, ACE, TCT-motif, and GT1-motif, were broadly present in the promoter regions. Notably, the G-box element was identified in most *TyubZIP* promoters (Figure 3B), and multiple copies were found in some genes, suggesting that *TyubZIP* may be extensively associated with light-responsive regulation. Considering the central role of HY5 in light signaling, we further examined the light-responsive cis-elements in the promoter region of *TyuHY5* and in two paclitaxel biosynthesis-related genes, *TyuDBTNBT* and *TyuTBT* (Table S4). Multiple light-responsive elements were identified in the TyuHY5 promoter. Notably, four G-box elements were detected in the promoter region of *TyuDBTNBT*, and multiple light-responsive motifs were also present in *TyuTBT*. These results provide a preliminary promoter-level basis for the possible involvement of *TyuHY5* in light-associated regulation of paclitaxel biosynthesis-related genes.

### 3.5. Genome-Wide Localization and Collinearity of TyubZIP Genes

**Figure 4:**
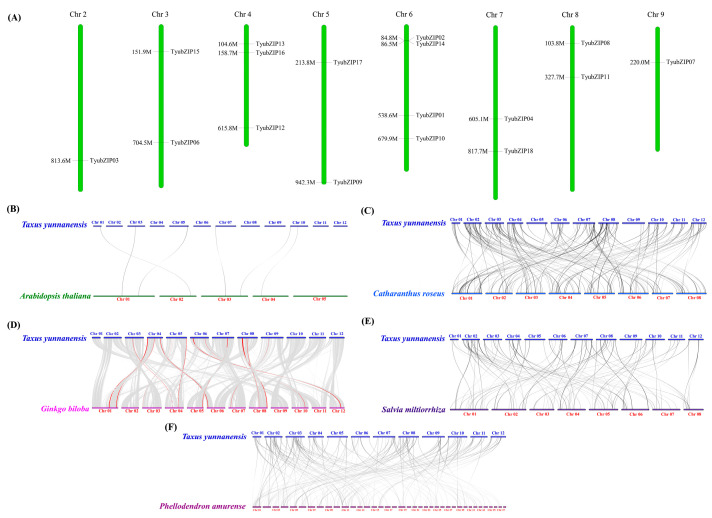
Chromosomal distribution and synteny analysis of *TyubZIP* genes. (**A**) Chromosomal locations of the 18 *TyubZIP* genes in *Taxus yunnanensis*. (**B**–**F**) Collinearity analysis of *bZIP* genes between *T. yunnanensis* and five representative plant species: *Arabidopsis thaliana* (**B**), *Catharanthus roseus* (**C**), *Ginkgo biloba* (**D**), *Salvia miltiorrhiza* (**E**), and *Phellodendron amurense* (**F**). Gray lines indicate all collinear blocks between species, and red lines indicate collinear bZIP gene pairs.

The chromosomal localization of *TyubZIP* genes was analyzed based on the reference genome (Figure 4A). The 18 *TyubZIP* genes were unevenly distributed across eight chromosomes (Chr2–Chr9). Among them, Chr4 and Chr6 each contained three *TyubZIP* genes, whereas Chr5, Chr7, Chr8, and Chr9 contained two genes each. Only one *TyubZIP* gene was identified on Chr2 and Chr3, respectively. No *TyubZIP* genes were detected on the remaining chromosomes. To further investigate the evolutionary conservation of *TyubZIP* genes, synteny analyses were performed between *T. yunnanensis* and four representative plant species, including *A. thaliana*, *C. roseus*, *G. biloba*, and *S. miltiorrhiza* (Figure 4B–E). The results showed that no clear collinear relationship was detected between *TyubZIP* genes and *bZIP* genes in *A. thaliana*, *C. roseus*, or *S. miltiorrhiza* (Figure 4B,C,E). In contrast, several collinear gene pairs were identified between *T. yunnanensis* and *G. biloba* (Figure 4D), indicating a relatively conserved syntenic relationship of *bZIP* genes between these two gymnosperm species.

### 3.6. Expression Profiles of TyubZIP Genes in Different Tissues and Under Shading Treatment

**Figure 5:**
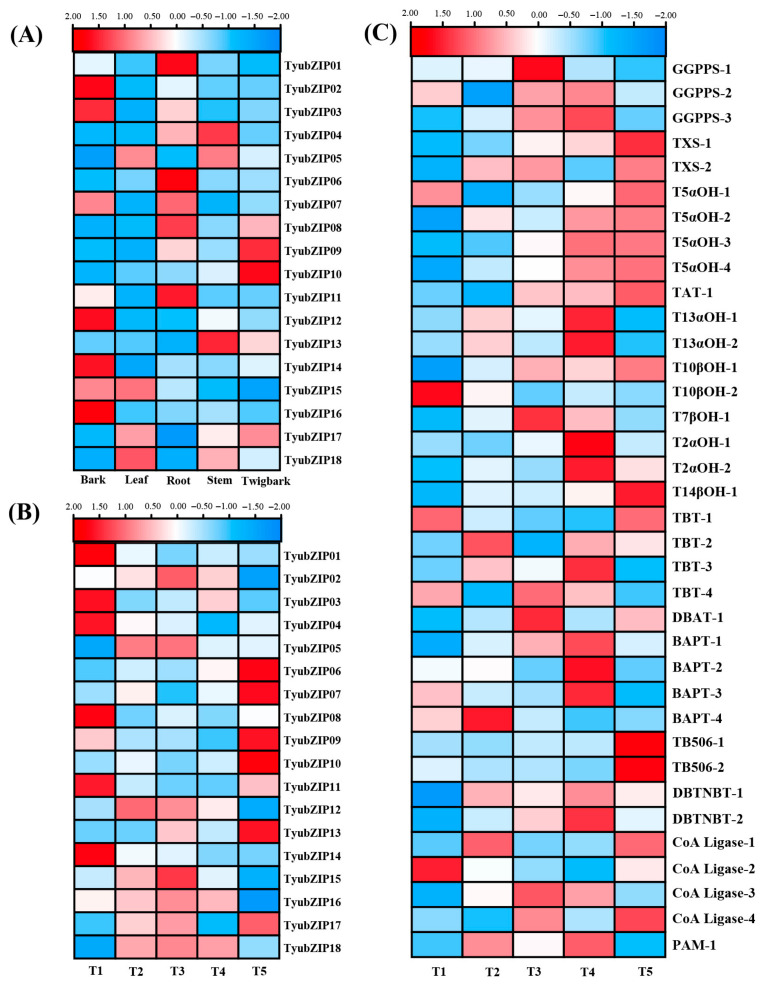
Expression profiles of *TyubZIP* genes in different tissues and under shading treatment. (**A**) Heatmap showing the expression patterns of *TyubZIP* genes in different tissues of *Taxus yunnanensis*, including bark, leaf, root, stem, and twig bark. (**B**) Heatmap showing the expression patterns of *TyubZIP* genes under different shading treatments (T1–T5). (**C**) Heatmap showing the expression patterns of taxol biosynthesis-related genes in leaf tissues under different shading treatments. Expression values are shown as FPKM, and the color scale represents relative expression levels across samples.

To characterize the expression patterns of *TyubZIP* genes, transcript abundance was analyzed in different tissues and under shading treatment (Figure 5A,B). The *TyubZIP* genes showed distinct tissue-specific expression patterns (Figure 5A). Several genes, including *TyubZIP01*, *TyubZIP02*, *TyubZIP03*, *TyubZIP06*, *TyubZIP12*, *TyubZIP14*, and *TyubZIP16*, were more highly expressed in bark or root, whereas *TyubZIP09*, *TyubZIP10*, and *TyubZIP13* showed relatively higher expression in twig bark or stem. Under different shading treatments, *TyubZIP* genes also exhibited diverse expression patterns (Figure 5B). Some members, such as *TyubZIP01*, *TyubZIP03*, *TyubZIP08*, *TyubZIP11*, and *TyubZIP14*, showed relatively high expression under T1, whereas *TyubZIP06*, *TyubZIP07*, *TyubZIP09*, *TyubZIP10*, and *TyubZIP13* were more highly expressed under T5. In addition, several genes displayed higher expression under intermediate light conditions, indicating differential responsiveness to shading intensity. To further examine the potential association between *TyubZIP* genes and taxol biosynthesis under shading treatment, the expression patterns of key taxol biosynthetic genes were analyzed (Figure 5C). These genes exhibited diverse expression profiles across T1–T5. Notably, *DBTNBT*, *BAPT*, *TBT*, and CoA ligase family genes showed marked expression changes under different shading conditions. Notably, *TyuHY5* showed relatively higher expression levels in bark and twig bark tissues, which are considered important sites for paclitaxel accumulation in Taxus species. Similarly, several paclitaxel biosynthesis-related genes, including *TyuDBTNBT* and *TyuTBT*, also exhibited elevated expression in these tissues (Figure 5C). The partially overlapping tissue-specific expression patterns between *TyuHY5* and paclitaxel biosynthesis genes further support the potential involvement of *TyuHY5* in the regulation of paclitaxel biosynthesis.

### 3.7. WGCNA Identifies Candidate TyubZIP Genes Associated with Taxol Biosynthesis

**Figure 6:**
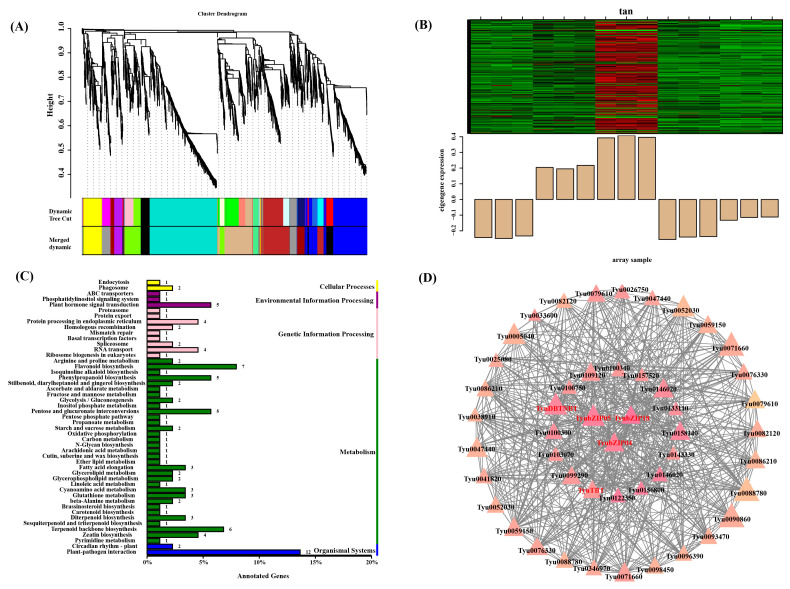
WGCNA-based identification of candidate *TyubZIP* genes associated with *TyuDBTNBT*. (**A**) Hierarchical clustering dendrogram of genes based on expression profiles. Different colors represent distinct co-expression modules identified by dynamic tree cutting and subsequent module merging. (**B**) Expression pattern of the tan module across samples. The heatmap shows relative gene expression levels within the module, and the bar plot below represents the corresponding module eigengene expression pattern. (**C**) KEGG enrichment analysis of genes in the tan module. The horizontal axis indicates the proportion of annotated genes, and the numbers beside the bars represent the numbers of genes enriched in each pathway. (**D**) Co-expression network of genes in the tan module. Nodes represent genes, and edges represent co-expression relationships. Node size and color intensity indicate intramodular connectivity (kWithin), with larger and darker nodes representing hub genes. Edge thickness reflects the strength of pairwise correlations between genes. *TyuDBTNBT* and candidate *TyubZIP* genes are highlighted in red.

To identify candidate *TyubZIP* genes potentially involved in taxol biosynthesis under shading treatment, a WGCNA was performed based on transcriptome data obtained from shading-treated samples. Hierarchical clustering of gene expression profiles identified multiple co-expression modules, which were further merged according to their expression similarity (Figure 6A). Among these modules, the tan module was selected for further analysis because its expression pattern was highly similar to that of key genes involved in the taxol biosynthetic pathway under shading conditions (Figure 6B). To further explore the biological functions represented in this module, KEGG enrichment analysis was conducted. The genes in the tan module were mainly enriched in pathways related to plant hormone signal transduction, protein processing in endoplasmic reticulum, proteasome, RNA transport, spliceosome, terpenoid backbone biosynthesis, diterpenoid biosynthesis, and plant–pathogen interaction (Figure 6C). These results indicate that the module is associated with multiple biological processes, including signal transduction, genetic information processing, and secondary metabolism. A co-expression network was subsequently constructed based on genes in the tan module (Figure 6D). In this network, *TyuDBTNBT* was closely connected with several genes, including multiple *TyubZIP* members. Notably, *TyubZIP04*, *TyubZIP05*, and *TyubZIP15* were located in the co-expression network surrounding *TyuDBTNBT*, suggesting that these genes may act as candidate regulators involved in the transcriptional regulation of *TyuDBTNBT*.

### 3.8. Functional Characterization of TyuHY5 and Its Regulation of TyuDBTNBT

**Figure 7:**
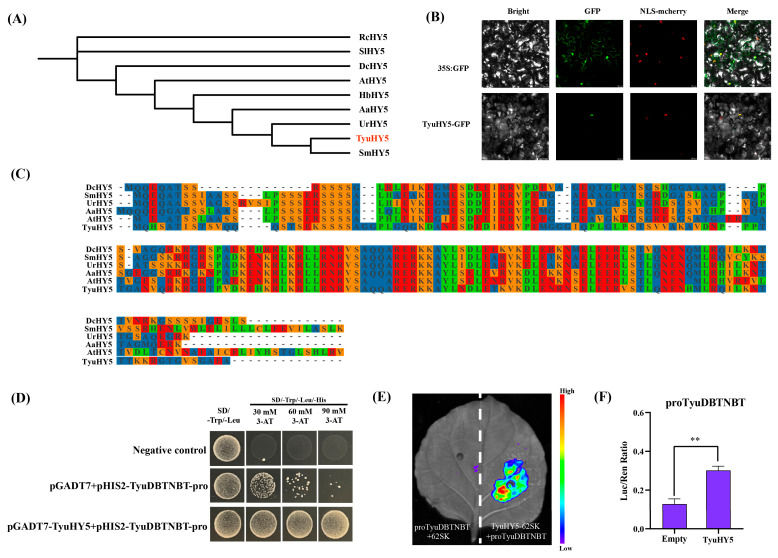
Functional characterization of *TyuHY5* and its regulation of *TyuDBTNBT*. (**A**) Phylogenetic tree of TyuHY5 and HY5 proteins from different plant species. (**B**) Subcellular localization of *TyuHY5* in *Nicotiana benthamiana* leaves. Bright-field, GFP, NLS-mCherry, and merged images are shown. (**C**) Multiple sequence alignment of TyuHY5 with HY5 proteins from other plant species. In panel (**C**), different colors indicate different amino acid residues in the multiple sequence alignment. (**D**) Yeast one-hybrid assay showing the interaction between *TyuHY5* and the promoter of *TyuDBTNBT*. (**E**) Luminescence imaging of *Nicotiana benthamiana* leaves in the dual-luciferase assay. (**F**) Relative luciferase activity expressed as the LUC/REN ratio. Data are presented as mean ± SD from three independent biological replicates (*n* = 3). Statistical significance compared with the empty vector control was determined using Student’s *t*-test (** *p* < 0.01).

Phylogenetic analysis showed that *TyuHY5* clustered closely with HY5 proteins from other plant species, particularly *SmHY5* and *UrHY5*, indicating that *TyuHY5* belongs to the conserved HY5 family (Figure 7A). Subcellular localization analysis further showed that the TyuHY5-GFP fusion protein was localized in the nucleus, whereas the GFP control was distributed throughout the cell (Figure 7B). Consistently, multiple sequence alignment revealed that TyuHY5 shared high sequence similarity with other HY5 proteins and contained the conserved bZIP domain characteristic of HY5 family members (Figure 7C). To investigate whether *TyuHY5* could interact with the *TyuDBTNBT* promoter, a yeast one-hybrid assay was performed. Yeast cells co-transformed with pGADT7-TyuHY5 and pHIS2-TyuDBTNBT-pro exhibited stronger growth on SD/-Trp/-Leu/-His medium supplemented with 30, 60, and 90 mM 3-AT compared with the negative control containing the empty pGADT7 vector (Figure 7D). Although weak background growth was observed in the negative control, the TyuHY5-containing transformants consistently displayed markedly enhanced growth, suggesting a potential interaction between *TyuHY5* and the *TyuDBTNBT* promoter in yeast. To further verify the regulatory effect of *TyuHY5* on the *TyuDBTNBT* promoter in planta, a dual-luciferase reporter assay was conducted in *N. benthamiana* leaves. Co-expression of TyuHY5-62SK with proTyuDBTNBT resulted in a much stronger luminescence signal than that of the empty vector control (Figure 7E). Consistently, quantitative analysis showed that the LUC/REN ratio in the *TyuHY5* group was significantly higher than that in the empty group (Figure 7F). Together, these results support the positive regulatory effect of *TyuHY5* on the promoter activity of *TyuDBTNBT*.

## 4. Discussion

Paclitaxel is one of the most important diterpenoid natural products in Taxus species, and although many biosynthetic enzymes have been identified, the transcriptional regulation of paclitaxel biosynthesis remains incompletely understood [9,46]. Transcription factors from several families, including MYB, bHLH, WRKY, AP2/ERF, and NAC, have been implicated in the regulation of specialized metabolism [47,48,49,50], while bZIP proteins are known to play important roles in development and environmental signal transduction [20,21]. In this study, we systematically identified 18 *TyubZIP* genes in *T. yunnanensis* and classified them into 10 subfamilies [47,48,49,50]. The predicted pI values of TyubZIP proteins ranged from 5.04 to 10.40, indicating substantial variation in protein charge characteristics within the family. Several TyubZIP proteins exhibited strongly basic properties (pI > 9.5), which may facilitate DNA binding and protein–protein interactions associated with their transcriptional regulatory functions [12]. Although the *TyubZIP* family size is smaller than that reported in many angiosperms, the major evolutionary lineages are conserved [51], indicating structural conservation alongside potential regulatory divergence among subfamilies. Conserved motif and domain analyses further suggest that, while overall structural features are maintained, differences among subfamilies may underlie functional specialization in regulatory networks. These findings provide a foundation for interpreting the shading-responsive expression patterns of *TyubZIP* genes and their possible roles in regulating paclitaxel biosynthesis [23]. By integrating phylogenetic, structural, and expression analyses, our results highlight candidate *TyubZIP* genes that may link light signaling to paclitaxel biosynthesis, offering a theoretical framework for future functional studies of these transcription factors. It should be noted that the lower number of *bZIP* genes in *T. yunnanensis* may reflect both a true biological reduction in this gymnosperm and the inherent limitations of the current reference genome assembly, which is large (~10 Gb), highly repetitive, and potentially fragmented. To further assess potential underestimation, we compared transcriptome data with the annotated *bZIP* genes and did not detect additional highly expressed bZIP-like transcripts outside the 18 identified genes, suggesting that the identified set likely represents the majority of *bZIP* genes in *T. yunnanensis* [2]. Conserved motif and domain analyses further showed that most TyubZIP proteins retained typical bZIP-related structural features, whereas differences in motif composition were observed among subfamilies, suggesting overall structural conservation accompanied by functional divergence [10]. Promoter analysis also revealed abundant cis-elements related to light responsiveness, hormone signaling, and stress responses, including G-box, ABRE, MYB, and MYC elements, implying that *TyubZIP* genes may participate in multiple signal-regulated transcriptional processes [52,53,54]. The synteny analysis revealed conserved *bZIP* gene pairs between *T. yunnanensis* and *G. biloba*, but no clear collinearity was observed with angiosperms. This pattern likely reflects the deep divergence between gymnosperms and angiosperms and may indicate lineage-specific expansion or loss of *bZIP* genes during gymnosperm evolution [2].

The expression analysis under shading treatment provided further evidence for the potential regulatory roles of *TyubZIP* genes. Light is a major factor affecting plant secondary metabolism, and increasing evidence shows that light-responsive transcription factors can regulate terpenoid and other specialized metabolic pathways [4,55]. In our study, *TyubZIP* genes exhibited distinct tissue-specific expression patterns and diverse responses to different shading conditions. Interestingly, *TyuHY5* and several paclitaxel biosynthesis-related genes exhibited relatively high expression levels in bark and twig bark tissues, which are known to be major sites of paclitaxel accumulation in *Taxus* species. This tissue-associated expression pattern further supports a possible role of *TyuHY5* in coordinating light-responsive regulation of paclitaxel biosynthesis. The widespread distribution of light-responsive cis-elements in their promoters, together with their differential expression under shading, suggests that members of this family may be involved in light-mediated regulatory networks in *T. yunnanensis* [19]. Because paclitaxel is a specialized diterpenoid, this result also implies a possible connection between light signaling and paclitaxel biosynthesis at the transcriptional level [4,47]. The WGCNA results further narrowed the candidate regulators potentially associated with paclitaxel biosynthesis. The tan module was selected because its expression pattern was highly similar to that of key paclitaxel biosynthetic genes under shading treatment, suggesting coordinated transcriptional regulation. KEGG enrichment showed that this module was associated with hormone signaling, terpenoid backbone biosynthesis, diterpenoid biosynthesis, and other biological processes related to signal transduction and secondary metabolism. In the co-expression network, *TyuDBTNBT* was closely associated with several *TyubZIP* members, providing an important basis for candidate gene screening. Since *DBTNBT* encodes a key enzyme in the downstream steps of paclitaxel biosynthesis, the identification of *TyubZIP* genes associated with *TyuDBTNBT* is biologically meaningful [56]. Although the present study experimentally validated the regulatory relationship between *TyuHY5* and *TyuDBTNBT*, WGCNA analysis also showed that *TyuHY5* was co-expressed with several additional paclitaxel biosynthesis-related genes within the tan module, suggesting that *TyuHY5* may participate in broader regulation of the paclitaxel biosynthetic pathway. However, these potential regulatory relationships still require further experimental validation.

In this study, phylogenetic analysis placed *TyuHY5* within the HY5-related clade, and motif/domain analyses showed strong structural conservation with other known HY5 proteins [52]. In addition, the nuclear localization of *TyuHY5* is consistent with its proposed role as a transcriptional regulator [48]. Combined with the promoter-binding and transcriptional activation assays, these findings suggest that *TyuHY5* may function as a positive upstream regulator of paclitaxel biosynthesis under shading-responsive conditions [57]. Previous studies have shown that paclitaxel biosynthetic genes can be regulated by multiple transcription factor families, including *bHLH*, *WRKY*, *MYB*, and *NAC* [57,58,59,60,61]. Our findings expand this regulatory framework by introducing a light-signaling-associated *bZIP* transcription factor into the paclitaxel regulatory network. Given the central role of *HY5* in integrating environmental signals with downstream metabolism, *TyuHY5* may serve as a molecular link between shading signals and paclitaxel biosynthesis. However, the present study mainly demonstrates its regulatory effect on *TyuDBTNBT* [21,22]. Although *TyuHY5* binds the *TyuDBTNBT* promoter and activates its transcription in heterologous systems (Y1H and *N. benthamiana* dual-luciferase assays), these systems do not fully reflect the native *Taxus* cellular context. *TyuHY5* expression varies with shading (Figure 5B), suggesting a shading-responsive role, but direct, sequence-specific binding in vivo remains unconfirmed. Future work including EMSA, site-directed mutagenesis, and chromatin-based assays will be needed to validate its regulation of *TyuDBTNBT* and other paclitaxel pathway genes, and to assess interactions with G-box elements and other signaling factors.

## 5. Conclusions

This study presents the first genome-wide identification and comprehensive characterization of the *bZIP* transcription factor family in *T. yunnanensis*. A total of 18 *TyubZIP* genes were identified and classified into 10 subfamilies. Analyses of phylogeny, conserved motifs, domain organization, cis-acting elements, chromosomal distribution, collinearity, and expression profiles revealed that the *TyubZIP* family is structurally conserved but exhibits divergence in regulatory characteristics and expression patterns. Expression profiling under shading treatment and WGCNA identified several candidate *TyubZIP* genes potentially associated with paclitaxel biosynthesis. Among them, *TyuHY5* was further characterized as a nuclear-localized HY5 homolog that can bind to and activate the *TyuDBTNBT* promoter in heterologous systems. Its expression varies with shading, suggesting a potential role in shading-responsive regulation of paclitaxel biosynthesis. However, its direct function under native conditions remains to be confirmed. These findings provide a theoretical basis for future functional studies of *TyubZIP* genes and offer insight into possible mechanisms linking light signaling to paclitaxel biosynthesis in *T. yunnanensis*.

## Data Availability

The genome sequences, protein sequences, and annotation files of *Taxus yunnanensis* and *Ginkgo biloba* were obtained from the 1K Medicinal Plant Genome Database (http://www.herbgenome.com/ (accessed on 1 May 2026)) and the Ginkgo Genome Database (https://ginkgo.zju.edu.cn/ (accessed on 1 May 2026)), respectively. The genome data of *Phellodendron amurense*, *Catharanthus roseus*, and *Salvia miltiorrhiza* were retrieved from Figshare (https://doi.org/10.6084/m9.figshare.27310872.v1), the Dryad Digital Repository (https://doi.org/10.5061/dryad.d2547d851), and the National Genomic Science Data Center (https://ngdc.cncb.ac.cn/ (accessed on 1 May 2026)) under BioProject no. GWHDOEA00000000. The transcriptome data of five tissues (bark, leaf, root, stem, and twig bark) of *T. yunnanensis* were obtained from the National Center for Bioinformation under BioProject no. PRJNA661543. In addition, the raw transcriptome sequencing data generated in this study have been deposited in the Genome Sequence Archive (GSA) database at the China National Center for Bioinformation (CNCB) (http://gsa.big.ac.cn/ (accessed on 1 May 2026)) under BioProject no. PRJCA043882 and accession number CRA028415.

## Supplementary Materials

The following supporting information can be downloaded at: https://www.mdpi.com/article/10.3390/cimb48050521/s1.
