# Supplementary figures and images for: Genome-Wide Analysis of bZIP Transcription Factors and Expression Patterns in Response to Shading Treatment in Taxus yunnanensis
Source: Curr Issues Mol Biol. 2026 May 17;48(5):521. doi: 10.3390/cimb48050521 (PMC13204714; doi:10.3390/cimb48050521)

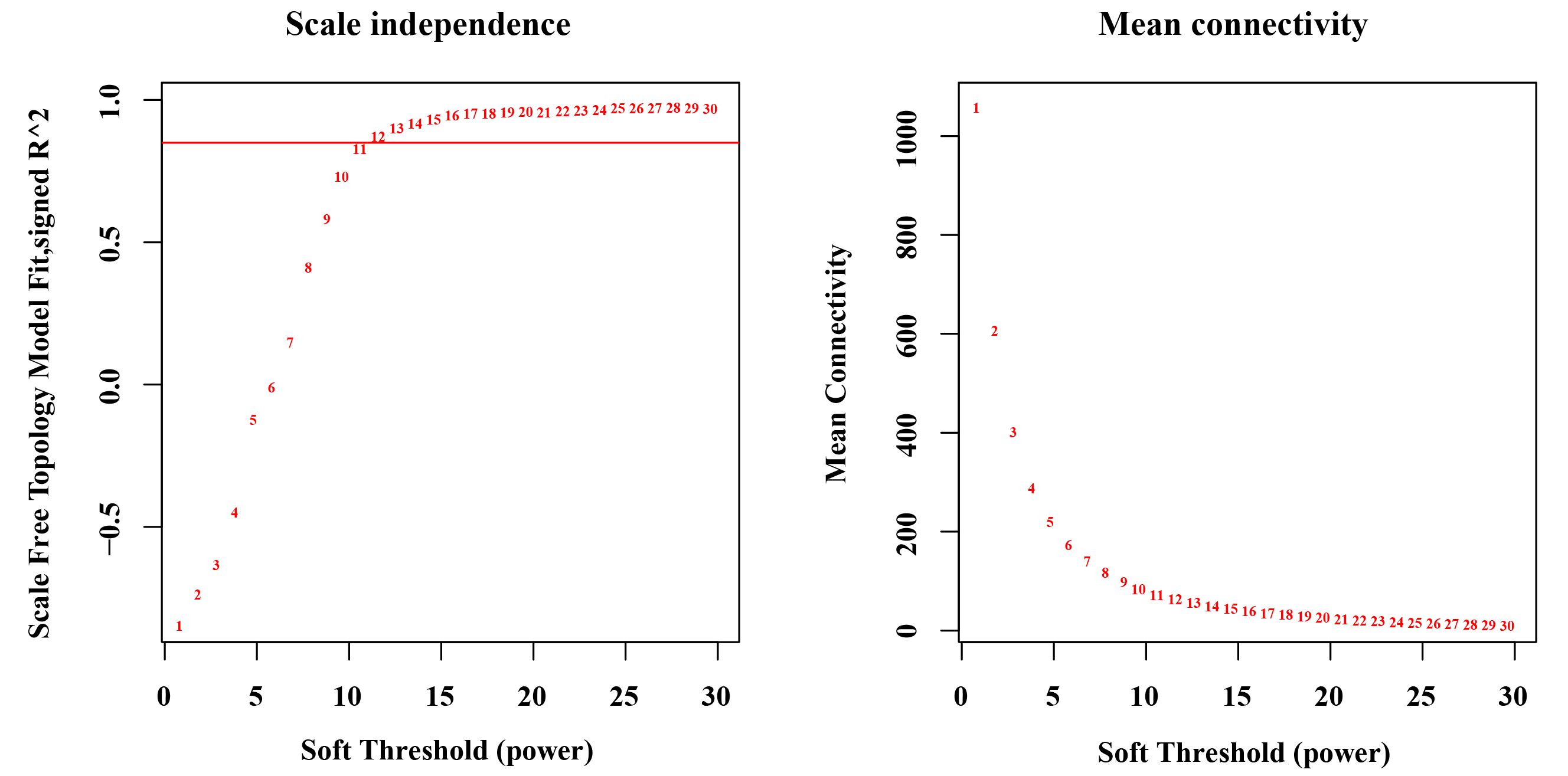

Supplement: Supplementary file 1 [file cimb-48-00521-s001.zip › Figure S1. Analysis of network topology for selecting the optimal soft-thresholding power in WGCNA..jpg]
